# Toward Polymeric and Polymer Composites Impeller Fabrication

**DOI:** 10.3390/polym14010097

**Published:** 2021-12-28

**Authors:** Nader Zirak, Mohammadali Shirinbayan, Michael Deligant, Abbas Tcharkhtchi

**Affiliations:** 1 Arts et Metiers Institute of Technology, CNRS, CNAM, PIMM, HESAM University, F-75013 Paris, France; mohammadali.shirinbayan@ensam.eu (M.S.); abbas.tcharkhtchi@ensam.eu (A.T.); 2 Arts et Métiers Institute of Technology, CNAM, LIFSE, HESAM University, F-75013 Paris, France; michael.deligant@ensam.eu

**Keywords:** impeller, polymers, polymer composites, manufacturing process, additive manufacturing, conventional manufacturing, injection moulding, performance

## Abstract

Impellers are referred to as a core component of turbomachinery. The use of impellers in various applications is considered an integral part of the industry. So, increased performance and the optimization of impellers have been the center of attention of a lot of studies. In this regard, studies have been focused on the improvement of the efficiency of rotary machines through aerodynamic optimization, using high-performance materials and suitable manufacturing processes. As such, the use of polymers and polymer composites due to their lower weight when compared to metals has been the focus of studies. On the other hand, methods of the manufacturing process for polymer and polymer composite impellers such as conventional impeller manufacturing, injection molding and additive manufacturing can offer higher economic efficiency than similar metal parts. In this study, polymeric and polymer composites impellers are discussed and conclusions are drawn according to the manufacturing methods. Studies have shown promising results for the replacement of polymers and polymer composites instead of metals with respect to a suitable temperature range. In general, polymers showed a good ability to fabricate the impellers, however in more difficult working conditions considering the need for a substance with higher physical and mechanical properties necessitates the use of composite polymers. However, in some applications, the use of these materials needs further research and development.

## 1. Introduction

Impellers are referred to as a key component of turbomachinery [1]. By rapidly rotating the impeller can force the working fluid by converting the velocity of the fluid to pressure [2]. Considering the use of impellers in different rotary machinery systems, they have played a vital role in various applications such as aerospace [3], automotive [4] and medical [5] applications. The improvement of a system’s efficiency by impellers has attracted attention in a lot of studies [6]. To this end, in general, the studies based on geometry optimization [7], the use of high-performance materials [8] and suitable manufacturing processes [1] have tried to improve the system. Weight loss of the impeller, along with the optimization of the impeller with a proper manufacturing process, can lead to achieving an ideal impeller. So that the use of lighter materials with high strength and an ability to withstand forces during working is considered as an effective step to increasing efficiency [9].

Metal [10], polymeric [11] and composite [12] materials are the main categories of materials that have been used for the fabrication of rotors and impellers. In general, the weight, high cost of raw materials, the fact that common methods of fabrication have led to increasingly high manufacturing costs, and the high maintenance cost of metallic rotors, have all proved to be main disadvantages of metals [10]. All these problems have led to polymers and composites being at the center of attention with regard to studies. Micro Organic Rankine Cycle (mORC), Heating, Ventilation, and Air Conditioning (HVAC) and refrigeration systems are among the systems which have the potential to replace their rotary components with polymer or polymer composites. For example, in the case of micro Organic Rankine cycle, which is one of the important systems for handling fossil fuel sources (flue gases and waste heat) and renewable energies, the replacement of metal rotors with polymer and composite rotors has been mentioned as an important factor in dealing with the limited use of these turbines due to their uneconomical cost [11].

Using polymers and composites due to low weight, good chemical resistance, and good strength have been a good choice for use instead of metals in the manufacture of rotors and impellers [13]. Also, the use of these materials in manufacturing can involve methods with lower costs compared to traditional methods for producing metal parts such as forging and casting. In general, thermoplastic polymers and thermoset polymers were among the polymers that have been used to fabricate the rotors. In thermoplastic polymers, acrylonitrile butadiene styrene (ABS) [11], polylactic acid (PLA) [14], polyether ether ketone (PEEK) [15] are the examples that have been used for fabricating the impellers. On the other hand, composites have been used in situations where a material with a higher strength is required [12]. Among the composites, PEEK-GF30 has been one of the composites that have been used for this purpose.

In this review, polymers and polymer composites that were considered for the fabrication of impellers in different applications were studied. Due to the importance of manufacturing methods and their effect on the final product, different manufacturing processes such as Additive manufacturing, Injection molding and Conventional impeller manufacturing were explained. Regarding, the importance of computational fluid dynamic for simulation and the interaction structural fluid, with respect to the main stresses, were discussed. Finally, performance tests for the evaluation of fabricated impellers were mentioned.

## 2. Materials Used for Fabrication of Impellers

Given the vital role of materials used in rotary components fabrication, with respect to the production of the different components and working conditions, the selection and manufacture of materials have been among the most important thing [4]. In this regard, great progress has been made in the materials used for the fabrication of impellers. As mentioned, metals, polymers, and composites have been among the main group of materials used to fabricate impellers. In general, in the metals class, stainless steel, titanium, aluminum and nickel alloys are among the metal-based alloys widely used to produce rotors, impellers and fans [16]. In high-temperature applications, such as a combustion chamber or turbine inlet, which are known as “hot zones”, Nickel alloys have been used. On the other hand, titanium alloys have been used in zones with lower operating temperatures, known as “cold zones”, such as compressor inlets and turbine outlets [3].

Considering the reasons mentioned, the focus of this study will be on polymers and polymer composites. Good resistance to impact loads [17], fatigue [18], erosion [19], and a high ratio between mechanical resistance and material density [9] are among the properties that are exemplary when using polymers and polymer composites in the production of impellers, and, as such, they have been a decisive choice. 

### 2.1. Polymeric Impellers

In different kinds of systems, in order to increase the efficiency of compressors and pumps and economic efficiency, polymers have been introduced for the fabrication of rotors [4,11,20,21]. Polymers defined as macromolecules consist of large numbers of smaller molecules, or repeating units, called monomers, which are formed chemically bonded. Polymer molecules can have a degree of order, relative orientation and a kind of monomer that can vary within the same polymer molecules [22]. Low price, ease of manufacture, resistance to water and versatility have been among the advantages of polymers, and these factors have led to their application in industry [23]. Polymers can exist in different forms of powders, granolas, filaments and resins, which are selected depending on the fabrication process. In general, polymers used for the fabrication of impellers can be divided in two categories of thermoplastic and thermoset polymers. 

#### 2.1.1. Impellers Based on Thermoplastics

**Figure 1:**
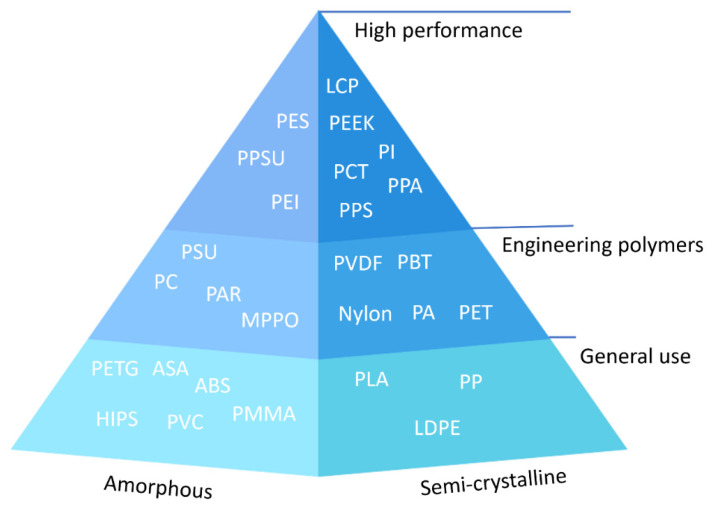
Different types of thermoplastics polymer.

Thermoplastics have been used to fabricate impellers in many studies. Generally, considering the ability of this group to be soften and melt when heated, two-state fabrication based on heat-softening or liquid state is preferable [24]. Injection molding [25] and 3-D printing-based processes such as fused deposition modeling (FDM) [26] or selective laser sintering (SLS) [27] are the methods by which thermoplastics are used for parts fabrication. Thermoplastics are divided in two groups of amorphous and semi-crystalline. Powders, granola and filament are different forms of thermoplastics polymers. Recyclability, good ductility and impact resistance compared to thermosets are the advantages of this class of polymer. In general, thermoplastic parts show a modulus lower than 5 GPa, which depends on the chemical composition and fabrication method as it can be changeable [24]. Figure 1 shows the different types of thermoplastic polymers with respect to ultra-performance, engineering-grade and general-purpose categories that represent the different classes of polymer materials.

Among the different types of thermoplastic polymers, ABS [11], PLA [14], Polyethylene terephthalate (PETG) [28], PEEK [15] and Polyphenylene sulfide (PPS) [29] are the examples that have been used for the fabrication of rotating components. Table 1 shows the physical and mechanical properties of these polymers. Modulus, cost, degradability, and water absorbability were the important parameters that impacted upon the candidate selection, and this concept will be discussed in Section 4.

ABS is an amorphous thermoplastic polymer that has been applied to the fabrication of rotary components in micro Organic Rankin Cycle (mORC) [11,36], pumps [13,37,38,39] and the rotor blades of drones [40,41]. Hernandez-Carrillo et al. [11] studied the use of ABS impeller in the mORC. This study was performed by increasing the efficiency of the mORC by reducing the conventional weight of the impeller. Working conditions of the impeller, such as inlet temperature and pressure were 55 °C, 4Bara and the outlet temperature and pressure were 44.9 °C, 2.5Bare, respectively. Also, the rotational speed was 36,000 rpm. Considering the working condition and factor of safety (FoS), which represents the ratio of yield strength per the maximum equivalent stress, the ABS impeller provided the expected operating condition. Reducing the cost of the fabrication of the impeller by using the ABS, which can cause the mass production of mORC, was one of the important advantages of applying this polymer. Also, according to their simulation results, with respect to the working fluid of Penta-fluoro-propane (R45fa), the isentropic efficiency of the impeller was estimated to be 76–86%. However, the limitated operation of ABS under 89.9 °C was mentioned as one of the narrow operation capabilities. Pavlovic et al. [37] investigated the mechanical properties of ABS in the fabrication of impeller pumps and showed that ABS can be considered as a good candidate for the fabrication of impellers. Polak [38] studied the ABS impeller for a radial centrifugal pump by verifying the hydraulic parameters. The results showed an increase in efficiency in rotational speed of 2950 rpm. The surface smoothness of the ABS impeller was mentioned as an effective parameter in increasing efficiency. 

PLA is a semi-crystalline thermoplastic polymer that is derived from renewable resources, such as corn starch or sugarcane. Biodegradability and composability of the PLA are among the properties of this polymer [42]. Economic cost, environment-friendly biocompatibility and suitable physicomechanical properties of this polymer have made this a suitable choice when compared to other polymers. PLA has been used for the fabrication of impellers for pumps [13,43,44], compressors [45] and marine [14] applications. For the fabrication of impellers in pumps and marine applications, PLA has been used. However, considering the accessibility of two polymers of PLA and ABS, these two kinds of thermoplastic have been compared in many studies as an impeller of the pumps. In general, considering the high level of brittleness of PLA when compared to ABS [37], it can be said that application by more stress ABS has been preferred. Birosz et al. [45] studied the PLA wheel for compressors given the importance of creep and orientation properties of the material, which are essential to designing the impeller of the compressor during rotation. Regarding tensile strength, creep and bending properties, these were considered when analyzing the PLA. Creep performance results showed that PLA creep behavior was most similar to the weakly cross-linked elastomer so that at low loads, the creep curve was held to a constant limit. According to their results, PLA was introduced as a material worth choosing when seeking long-term service.

PETG is an amorphous thermoplastic [46]. PETG impeller has been used in pump [47,48] and mORC [36] applications. Good water-resistance and biodegradability of this polymer [49] are reasons for choosing this polymer in the manufacture of pump blades. Odetti et al. [48] investigated the PTEG impeller in the application of a Pump-Jet Module (PJM). Considering the rotational speed of 1200 rpm that led to a thrust of 14 N, a PTEG impeller showed suitable properties during the working for this application. 

Polyether-ether-ketone (PEEK) is a high-performance semi-crystalline thermoplastic polymer [35]. Excellent mechanical and thermal properties, as well as good chemical resistance, are among the bold advantages of this polymer [50]. PEEK impeller has a high position in pump and compressor applications for different industries such as automotive [51], aerospace [52] and medical [5]. In the case of medical applications, using the PEEK impeller in centrifugal pump due to the improved durability and strength it offers has attracted a lot of studies’ attention [5,53,54]. Similarly, in the case of heart failure, using the PEEK impeller in the centrifugal pump of a HeartWare Ventricular Assist Device (HVAD) due to the improved durability and strength it offers has attracted a lot of studies’ attention. In the HVAD, the rotational speed of the PEEK impeller is in the range of 1800–4000 rpm and generates flows up to 10 l/mL [5]. Also using the PEEK impeller with the aim of reduction in wear, reduced noise levels and more consistent running properties by replacing stainless steel for regenerative pumps was another application of this polymer [51]. In general thermomechanical properties, due to the thermal stress during increased temperature, is one of the important factors that can lead to the limitation of using the polymers as impellers in various applications. 

Zywica et al. [15] studied the use of plastics with the aim of using them as an impeller for the ORC system. In this study, PPS and PEEK were considered as thermoplastic polymers. The rotational speed for the impeller was 120,000 rpm. The simulation results based on heat resistance, chemical resistance, strength properties, and thermal expansions showed that PEEK polymer can be considered as a good material for the fabrication of impellers.

#### 2.1.2. Impellers Based on Thermosets

Thermosetting polymer can be defined as a soft solid or viscous state prepolymer that can be changed to the infusible, insoluble polymer network (thermoset) by curing. Curing of the prepolymer can be performed based on heating or suitable radiation. During the curing, cross linking the materials leads to them setting and they can no longer flow [55]. The main components of thermosets consist of monomers, co-monomers (hardeners), catalysts and initiators. Also for improving the mechanical properties and reducing the costs, some fillers such as calcium carbonate, sawdust, recycled powdered thermosets, etc., can be used in the formulation of thermosets [56]. In addition, the use of the short fiber to improve the mechanical properties is one of the important ways to increase the mechanical properties [57], which will be discussed in the composite section. Thermosets are divided into epoxy resins, phenolic resins, amine–formaldehyde, polyurethanes, silicones, cyanates, vinyl esters, dicyclopentadiene and other metathesis thermosets. Depending on the different formulations, different physical and mechanical properties can be achieved from the thermosets. For example, glass transition temperature can vary in the range of 20 to 200 °C [58]. Modulus can be achieved for light-cured resin [59] and continuous carbon fiber reinforced thermosetting composites [60] in the range of 0.18 and 161.4 GPa, respectively. Thermoset manufacturing processing is divided into categories: additive manufacturing techniques; solid thermoset processing; and liquid thermoset processing [61]. 

**Figure 2:**
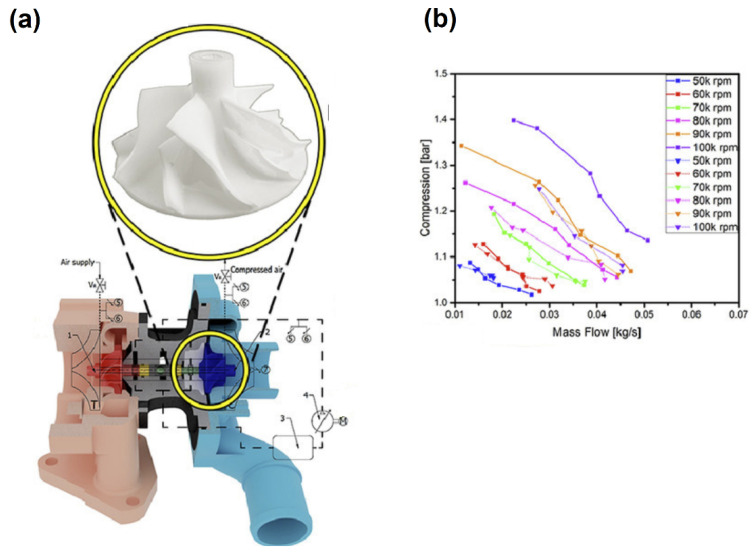
(**a**) Cross section of turbocharger with compressor wheel fabricated by MJP and (**b**) experimental results of polymeric (dotted lines) and aluminum (solid lines) compressor wheels (reprinted with permission from [9]).

Matveev et al. [62] studied the thermoset impellers fabricated by the SLA method. High chemical resistance, practically inert to liquid hydrocarbons (gasoline, kerosene, petroleum and synthetic oils) and hot streams (up to 100 °C) of water and air fabrication were some requirements of their study. By their study, thermoset impellers fabricated by 3D printing were considered as parts that fully meet the requirements of the experimental samples for gas-dynamic studies. In the case of the turbocharger, Andrearczyk et al. [9] investigated the wheel printed by a thermoset. The size of rotor was 42.5 mm in diameter (Figure 2a) and the maximum rotational speed was selected at 100,000 rpm. According to the simulation results, the maximum stress on the impeller for a rotational speed of 90,000 rpm was 27 MPa, whereas the yield stress of the resin printed was 54 MPa. We should mention that the results obtained from the compressor wheels, which were fabricated by polymer and aluminum, showed that at 90,000 rpm the polymeric wheel can operate like an aluminum wheel (Figure 2b).

### 2.2. Polymer Composites Impellers

The key role of composite materials, considering their weight reduction effect without sacrificing robustness, has been shown in the modern industry [63]. In this section, only the polymer matrix composite will be discussed. In the polymer matrix composite, the mechanical properties of the materials will be improved by using the fiber as reinforcement in the matrix of the polymer. Considering the type of matrix and reinforcement, the composites can have different categories. In general, reinforcement fiber can be divided into inorganic, glass and carbon fibers [64], and composite reinforced with either glass fibers (GF) or carbon fibers (CF) have been included in more than 90% of the studies [65]. Thermoplastic [66] or thermoset [67] polymers have been used as polymeric matrices for fabrication of impellers.

#### 2.2.1. Carbon Fiber as Reinforcement in Fabrication of Impellers

Carbon fiber polymer-matrix composites have been introduced as one of the efficient classes of material, in the place of metals. Depending on the type of fiber condition, be it short or continuous, these types of composites can be classified. PPS, PEEK, PI and PEI are the thermoplastics and epoxy is the thermoset, which has been widely used as a matrix for these composites [68]. PEEK [52,69] and epoxy [69,70], carbon-fiber-reinforced, are the composites that have been most used in the fabrication of impellers. 

PEEK reinforced with carbon fibers has been one of the exemplary composites used in the fabrication of rotary components of pumps and compressors [71,72]. PEEK composites reinforced with polyacrylonitrile short carbon fibers, 30% in weight, which is called CF30 PEEK is a famous commercial type of this composite [73]. Garcia-Gonzalez et al. [74] investigated the energy absorbed to analyze the mechanical impact behavior of short carbon fiber reinforced PEEK composites and unfilled PEEK. Tensile elastic modulus of GF30 PEEK in transversal, longitudinal conditions and unfilled fiber PEEK were 12.6, 24 and 3.6 GPa, respectively. According to their results, reinforced composites showed a brittle failure. The direction of fibers and degree of crystallization played a key role in the mechanical properties. The homogenization of elastic material and anisotropic damage for failure prediction has been proposed in their study. Investigating the vapor-grown carbon nanofibers for use as reinforcement for PEEK showed that by increasing the nanofiber, the modulus of composites increases, which refers to the effect of fiber on the crystallization of PEEK. Also, the effect of carbon nanofiber as a lubricant, which was associated with significant decreases in the wear rate of the composite, was shown [75]. Yang et al. [76] studied the effect of the surface modification of carbon fiber on the mechanical properties of CF PEEK composites. Their method was introduced as one of the main solutions to enhancing the interface and led to reaching an interfacial shear strength of 83.13 MPa. 

The investigation of the PEEK carbon-reinforced impeller in the case of a micro-turbine-generator introduced this composite as a suitable material instead of an aluminum impeller [11,36]. We should mention that the mechanical properties of the impeller at the rotational speed of 32,040 and 40,500 rpm were appropriate. However, considering the importance of the chemical reaction of working fluid with impeller in ORC and refrigeration systems, the final approval of the substance was considered dependent on more studies [36]. Martynyuk et al. [12] investigated using the polymer reinforced with carbon fiber for the fabrication of a centrifugal compressor wheel. The maximum working temperature was 287 °C, and the outlet pressure was 7 bar. The calculations showed that carbon fibers UMT 49S and phthalonitrile binder PN-3M, which have been used as a reinforcing part, can be used to fabricate the wheel of a centrifugal compressor. Also, the use of composite resulted in a 45% reduction in rotor weight compared to the similar aluminum specimen. 

Using the carbon fiber in the matrix of thermosets (especially epoxy) to improve the properties, has been used in order to fabricate impellers in pump and compressor applications [67,68,70]. In general, resisting moisture and other environmental influences, offers lower shrinkage and better mechanical properties are among the points that lead to the selection of epoxy resins as a polymeric matrix [77]. Shah et al. [78] studied the thermo-mechanical characterization of different types of epoxy resin of HinpoxyC, HinpoxyVB, ARL135 and ARL136 epoxy resin systems reinforced by HCU200/A45 carbon fiber. Tensile strength of the reinforced epoxy resin systems of HinpoxyC, HinpoxyVB, ARL135 and ARL136 were achieved 745, 752, 698 and 830 MPa, respectively. In general, the final properties can be variable depending on the type of matrix, carbon reinforcement, and fabrication process. For example, Ming et al. [79] studied the different parameters in 3D printed continuous carbon fiber reinforced thermosetting epoxy, such as printing speed, printing space, printing thickness, curing pressure and curing temperature with the aim of optimizing the parameters. According to their results, optimized conditions with 58 wt.% fiber led to them achieving the maximum flexural strength and modulus of 952.89 MPa and 74.05 GPa, respectively. Furthermore, Pérez-Pacheco et al. [80] studied the effect of moisture absorption on damage accumulation in carbon fiber–epoxy composites laminates with respect to the two different superficial carbon fiber treatments. In their studies, the interphase microstructure has been mentioned as a critical aspect of the moisture diffusion mechanism. Considering the number of hydrogen bonds between the water and epoxy resin network, and the two different activations of energy, subsequently, different phenomenon such as swelling or degradation can happen. Also, the sensibility of matrix failure mechanisms caused by hydrolysis has been discussed.

Uhlig et al. [81] studied the highly stressed bladed rotor fabricated by epoxy resin and reinforced with 60% carbon fiber. According to their results, the explosion rational frequency of the rotor was in the range of 1080–1100 Hz. Also, stress exposure factor for the fiber fracture at the explosion frequency range was about 0.8, so that this factor for the interfiber fracture should not have exceeded the failure limit of 1. The composite rotor has been considered as a suitable candidate to improve the efficiency compared to aluminum alloy rotors. Liu et al. [82] used the carbon fiber reinforced shape memory epoxy composites to fabricate the wind blades. The stiffness under good shape memory fixation at room temperature and switching temperature (Tsw) reached 37 and 4.4 GPa, respectively. According to their results, a sustainable continuous stable mechanical state has been observed. Also, variable wind speed in the range of 9–10 m s^−1^ was provided. 

#### 2.2.2. Glass Fiber as Reinforcement in Fabrication of Impellers

Composites of the polymeric matrix reinforced with glass fibers have been used for the fabrication of impellers [83]. In general glass fiber is an inorganic non-metallic material. Heat resistance, high tensile strength, and excellent chemical stability are among the properties of these fibers [64]. The composition of the glass fiber consists of SiO_2_, Al_2_O_3_, TiO_2_, B_2_O_3_, CaO, MgO, Na_2_O, K_2_O and Fe_2_O_3_. Differences in the composition of fibers lead to the appearance of various properties, which puts them in different categories. Different Young’s modulus can be achieved in the range of 51.7 to 86.9 GPa [82,84]. 

Fan et al. [85] investigated the diffusion of water in glass fiber reinforced polymer composites, with respect to room temperature and 50 °C. According to the micromechanics model, moisture diffusion in the GFRP in deionized water for matrix at room temperature and 50 °C were 1.41 × 10^−7^ and 4.57 × 10^−7^ mm^2^/s, respectively. Also, there was good agreement between the model and the experimental data. The diffusivity of fluid in the GFRP composites in the fiber was smaller than that of the polymeric resin, however it was not negligible. In such a way, the lateral fiber diffusivity was determining the factor that would control the thickness diffusivity of the GFRP plates. Nayak and Ray [86] investigated the residual mechanical properties of nano-Al_2_O_3_ filled glass fiber reinforced polymer composites (nano-GFRP) in the hydrothermal environment. According to the results, the addition of 0.1 wt.% of nano-Al_2_O_3_ into the GFRP composite has reduced the moisture diffusion coefficient to 10%. However, this addition has led to the improvement in the residual flexural and interlaminar shear strength by 16 and 17%, respectively, compared to the GFRP. They showed that nano-GFRP has created an opportunity to use this composite in a hydrothermal environment. Improvement in the wear properties of composites reinforced with glass fiber is another important factor that attracted the attention of a lot of studies [87,88]. Öztürk et al. [89] analyzed the erosive wear behavior of the epoxy resin and the glass-fiber-reinforcement was evaluated with respect to the different parameters, such as various impingement angles (from 20° to 90°), velocity in the range of 70–200 mm/s, exposure time and erodent size. According to their results, the best erosion resistance has been achieved for composite filled with 16 wt.% silica fume. 

Umaras et al. [29] studied the impeller fabricated by reinforced PPS with 40% fiberglass in the application of an automotive water pump. The rotational speed was 4500 rpm, and the working fluid was water and ethylene-glycol in the temperature range of 80–100 °C. Maximum radial and tangential stresses due to the press fit on the impeller have been calculated 100 and 83.5 MPa, respectively. Considering the physicomechanical properties of PPS reinforced with glass and maximum stress on the impeller this material has been considered a suitable selection in this situation. In the case of a micro-turbine-generator, PEEK-GF30 radial turbine impeller has been considered as one of the suitable candidates. Isaias et al. [36] investigated the fabrication of a micro-turbine-generator for an Organic Rankine Cycle (ORC) with the aim of replacing polymers with metals, and they considered the high technical and economic potential of polymers. For this purpose, the rotor was produced by the FDM process by using polymer and composite materials and the diameter of impeller was 45 mm. The results showed the ability to rotate the rotor at a rotational speed of 32,040 rpm and a peak rotational speed of 40,500 rpm. Also, due to the importance of the final surface obtained for the rotors and impellers on the final efficiency [90], it was shown that an acceptable final surface has been obtained by this method for the fabricated rotor. In another study, Organic Rankine Cycle microturbines fabricated by aluminum, ABS and PEEK-G30 were compared together. The diameter and rotational speed of the impeller were 49 mm and 36,000 rpm and the R245fa fluid was used as a working fluid. According to their study, in addition to the fact that the PEEK-GF30 and ABS showed they were suitable for mass production processes, the economic benefits, properties such as chemical resistance and lower inertia, with the latter characteristic helping to minimize imbalance, shaft fatigue, and damage of the casing in case of failure, were all among the other advantages they found. In addition, results of a simulation showed that PEEK-GF30 and ABS can be good candidates in these operating conditions and are good alternatives to aluminum in this application [11].

In general, required properties for working conditions, economic efficiency, and manufacturing methods are among the parameters that can impact the selection of polymers or polymer composites to fabricate the impellers. Also, recyclability of the materials, and any ecological problems the present, are also important parameters to consider when choosing the materials. So, thermoplastics can show more compatibility to this end, compared to the thermosets and polymer composites.

## 3. Manufacturing Process

The important effect of the manufacturing method can be clearly be linked to the energy consumed during the process and impeller performance [1,3]. The ability to be mass produced, attainability of suitable mechanical properties, a good surface, high precision for complex geometries, and economic efficiency are among the criteria to be considered when selecting the fabrication process of impellers. Additive manufacturing, milling and injection molding have been among the methods used to fabricate the polymeric and composite impellers.

### 3.1. Conventional Impeller Manufacturing

Conventional impeller manufacturing has consisted of a process in which the impeller is fabricated through the machining processes in a subtractive way. In this way, by removing additional layers, the desired shape with different accuracy will be manufactured depending on the selected machining and parameters process. Turning, milling, drilling and grinding are among the conventional manufacturing which can be applied for machining polymeric and composite materials [91]. 

Mainly, parameters that impact the final polymer or composite machined product can be divided into three categories: machine and environmental variables; tool design and machining conditions; and composition of the substances. The machine and environmental parameters such as slide straightness, temperature stability and vibration are the general parameters that controlled the dimension on a large scale. Surface roughness and delamination factor have been the effect by tool design and machining conditions such as rake angle, tip radius, depth of cut and cutting speed. Another important parameter referred to is the composition, which depends on the different physical and chemical properties as machinability will be variant. Also when comparing the polymers and composites, polymers are more homogeneous and have been accompanied by better machining capabilities. Delamination, cracking, fiber pull-out, and burning are among the defects which can happen during composites machining.

Among the different machining process, milling has been severally applied to the fabrication of polymeric and composite impellers [11,36,92,93]. The most common milling machining can be divided into the peripheral milling or profiling and end milling. In this method, extra material will be removed by rotating a cutterhead with control based on computer numerical control (CNC) which is called CNC milling [94].

Hernandez-Carrillo et al. [36] investigated the PEEK-GF30 impeller fabricated by five-axis CNC milling. According to their study, fabricated impeller showed good surface and acceptable mechanical properties against the centrifugal force load. Mentzos et al. [92] investigated the polymeric impeller fabricated by the CNC milling process in a pump application. Effect of process parameters such as cutting speed, feed rate and depth of cut has been considered in the final roughness surface of the impeller. Their results showed that by reducing the tool step-over and feed rate a smoother surface was obtained.

### 3.2. Injection Molded Impellers

Injection molding consists of four-steps of the cyclic process that includes the phases of filling, packing, cooling, and ejection to fabric the parts. Granola, or powder under pressure, and temperature will be molted and used to fill the mold. Depending on several parameters, such as raw materials, mold design, and process-specific parameters the final quality of the parts can be different [95]. Cost reduction and production in a short time have been among the parameters which attracted the attention of studies to fabricate the injected impellers [96,97]. In general, the cost of fabricated parts can be estimated by the parameters such as mold base, number of cavities, and injection mold. As to increasing the performance of the hydraulic pump from the injected impeller, adjusting the distance of the front shroud and rear shroud, namely the impeller outlet width, has been the most economical way to increase efficiency through the injected impeller [29,96,98]. 

Process parameters of injection molding such as molding temperature, melt temperature, injection pressure, and injection time have been introduced as important parameters of the properties and cost of fabricated parts [99]. The optimization of this parameter for the fabrication of the injected impellers attracted the attention of a lot of studies. 

Rosli et al. [100] have studied the optimization of process parameters of injected blower impeller fans with respect to the melting temperature, molding temperature, injection time and injection pressure processing parameters. Polypropylene has been used to fabrication the impellers. Given that in the response surface methodology the results optimum of mold temperature, melt temperature, injection time and injection pressure have been 110 °C, 210 °C, 0.8 s and 212.81 MPa., Shen et al. [101] has investigated the mold cooling design optimization for fabrication of the injected impeller. The melting temperature of the polymer was 230 °C. According to their study, the maximum ejection temperature of the impeller had reduced from 53 °C to 33 °C with a conformal cooling channel. Also, their mold had provided a higher cooling efficiency and a more uniform cooling. Topology optimization to reduce the mass of the mold showed the total mass reduction was about 20%.

### 3.3. Additive Manufacturing (AM) in Fabrication of Impellers

Additive manufacturing (AM) is a manufacturing process based on the fabrication of parts by joining the materials, directly from the 3d model. Since 1980 some sources have fabricated part of what was produced directly by a suitably formatted data file in a layer-by-layer fashion, and this can therefore be considered as the start date of AM processing. Over the past years, due to the significant increase in mechanical properties obtained by the production of AM, this method has been introduced as a desirable and reliable method for the production of parts. AM technology can be classified to the liquid polymer, discrete particle, molten material and solid shield systems. The use of AM to rotor and impeller fabrications is no exception and has attracted the attention of many studies. Selective laser melting (SLM) [102,103], electron beam melting (EBM) [104,105], stereolithography (SLA), fusion deposit modelling (FDM) and MultiJet printing technique (MJP) are the AM methods which have been used for rotor and impeller fabrications.

**Figure 3:**
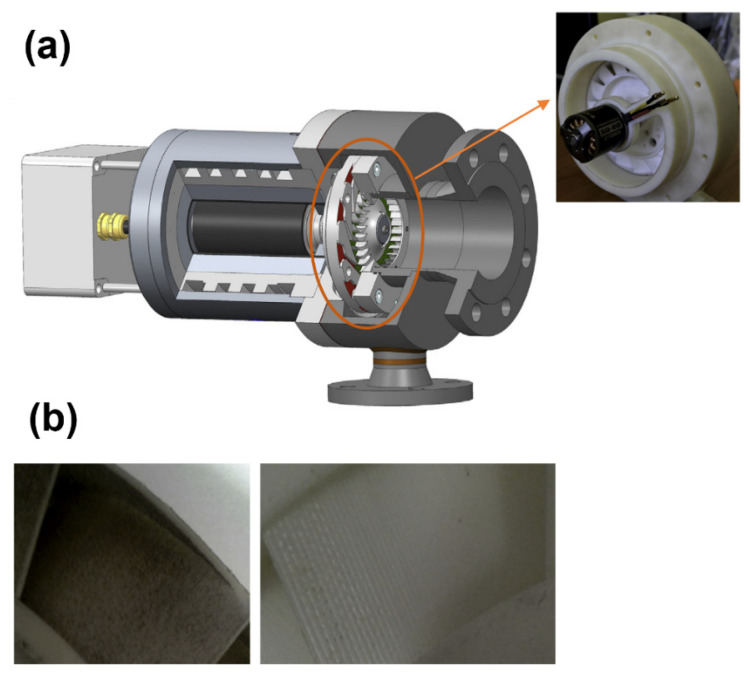
(**a**) Schematic of developed radial quasi impulse cantilever design and (**b**) left: fabricated rotor by SLS right: fabricated stator by FDM+SLS (reprinted with permission from [111]).

One of the most common and low-cost methods among the AM process is FDM. In this method the melting of the thermoplastic filament from a nozzle at a certain speed means parts can be prepared [106]. In several studies FDM has been used to produce the compressor and pump impellers. Quail et al. investigated the pump impeller by FDM method. Caille et al. [107] studied the pump impeller fabricated by FDM method. The impeller was 75 mm in diameter and 1.3 mm in thickness. The impeller was fabricated for use in a pump by a 3-kW induction motor and rotational speed of 3000 rpm. According to their results the impeller produced by this method was ensured to show qualities that included strength, performance and speed of manufacture. In a study by Fernandez et al. [13] FDM-impeller fabricated for pump purpose has been analyzed. Their study showed that, the impeller fabricated by FDM had a similar performance to the original impeller of the rotodynamic hydraulic pump. Also, considering the important effect of impeller roughness on the performance of compressors and pumps, the inherent roughness of the external impeller surfaces had no limitation in the results of the head-flow curve of the pump. In another study by Priyanka and Varaprasada Rao [108] they concluded that an impeller produced by FDM can be introduced as a deserving method, which can replace traditional manufacturing techniques in the industries. However, the difference of mechanical properties in various directions (anisotropy) has been one of the problems that have always existed in parts made by the FDM process [109]. Badarinath et al. [110] investigated the development and characterizes the performance of a robotic FDM system instead of FDM machines based on the three-axis cartesian system. They showed that an impeller fabricated by this method had regions of infill and perimeter overlap in the base and was free from voids and over-deposition. The results also indicated that a uniform deposition at regions of directional changes have parts with complex geometry. This method can be used for the fabrication of the parts with more complex geometries. Weiß et al. [111] studied the potential of additive manufacturing for the fabrication of different components of the ORC system by introducing a micro-turbine-generator-construction-kit (MTG-c-kit) in a customized turbogenerator (Figure 3a). As such, the air turbine was fabricated at 120 mm in diameter. Selective Laser Sintering (SLS) and FDM were the additive manufacturing methods used for the fabrication of this turbine through the Nylon and ABS, respectively. In this study, achieving a good surface quality of fabricated rotor was mentioned as an important parameter that can impact performance. The surface quality of the fabricated rotor through the SLS and FDM is shown in Figure 3b. According to their results for the fabricated wheel with Nylon by SLS, the first performance of the system was around 10%, which with a sufficient sealing of the surroundings of the stator nozzles increased to 20%. This maximum efficiency was achieved at the design pressure ratio and rotational speed of 1.4 bar and 6000 rpm, respectively. Also considering the better surface quality of the FDM compared to the SLS, the performance in the pressure ratio of 1.6 was similar. We should be mention that the notches on the surface of the FDM rotor were perpendicular to the airflow.

Stereolithography (SLA) is a technique where the polymerization of a photocurable liquid monomer in a spatially selective manner occurs using an ultraviolet light (or a laser). The 3D structure is achieved by alternating between the thin liquid films and spatially controlled photopolymerization steps [112]. Generally, rotors or impellers fabricated by this method are used in two goals: directly used as a rotor or impeller, or used in casting methods [113]. In an industrial case, Przybylski et al. [114] investigated the SLA-impeller fabricated for pump. The pump rotor, which was in the medium range size, analyzed the pump at the nominal load and at 3000 rpm rotational speed. According to their study, the rotor produced by this method had acceptable results compared to rotors that were commonly used. 

In the study by Isaias et al. [11], the AM method was applied for the fabrication of an impeller for the Organic Rankine Cycle (ORC) radial microturbine. In this study, a thermoplastic impeller (ABS) with a diameter of 49 mm was used for rotation at a speed of 36,000 rpm. The results of their study showed that the rotor made by this method could compete with the aluminum sample. In addition, the latter characteristic helped to minimize imbalance, shaft fatigue, and damage of the casing in case of failure and were among the advantages achieved by this method. 

**Figure 4:**
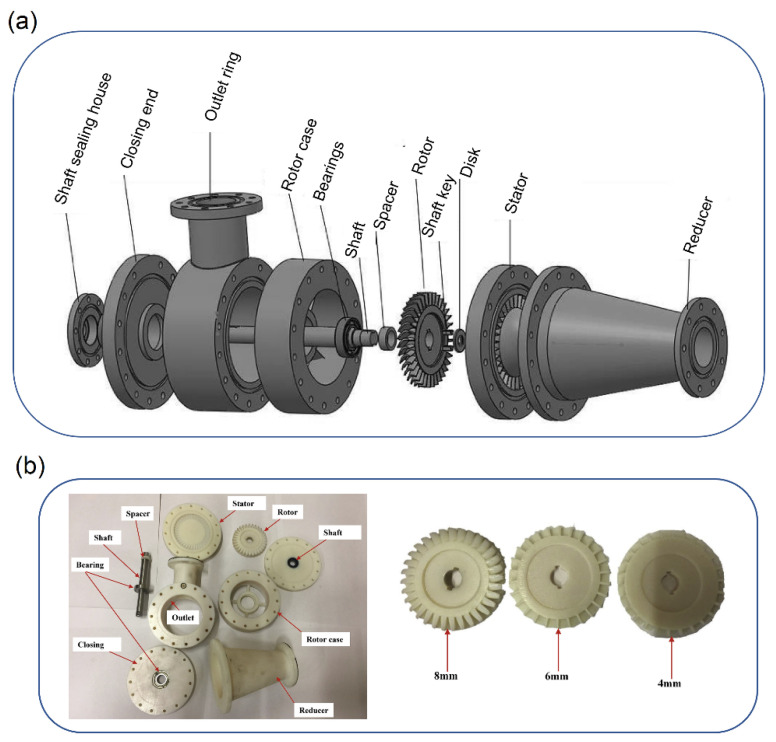
(**a**) Exploded schematic of micro-scale axial turbine and (**b**) left: fabricated all turbine parts and right: fabricated rotors with different blades heights (reprinted with permission from [118]).

Inkjet printing (IJP) can be defined as a technology for printing by depositing tiny droplets onto a substrate without dependence on the high-speed operation of mechanical printing elements. Polyjet and multijet printing are famous processes based on this technique [115]. In this method photocurable resin by a piezo printed process can be layer by layer fabricated. The high precision of this method to fabricate the complex geometries is one of the highlight features of this technique. Studies have introduced this technique as a high-performance method for making rotors and impellers [116]. Andrearczyk and Żywica [117] fabricated the compressor wheel and turbine wheel by MJP with the purpose of using them in the turbocharger. According to their study, the pressure range of the compressor was in the range of 0 to 1.6 MPa. Also, considering the tensile strength of this photopolymer, which mentioned around 65 MPa, and the working temperature of 88 °C, this process by this type of photopolymer was an assured method to fabricate the mentioned parts of the turbocharger. A study by Artur Andrearczyk et al. [9] using the MJP investigated the range of applications of this method concerning design, testing, and optimization of the elements of fluid-flow machines. In this study, the polymeric fabricated rotor was in the range of 42.5 mm, which was applied in the compressor inlet of the turbocharger (turbochargers are the fluid-flow machines with one of the highest nominal rotational speeds). The temperature range was 50 to 150 °C for the inlet air of this machine. The rotational speed up to 100,000 rpm was analyzed in this experiment. We should mention that the results obtained from the compressor wheels, which were fabricated by polymer and aluminum, showed that at 90,000 rpm the polymeric wheel can operate like the aluminum wheel. While the tensile strength and glass transition of the standard experiment of this polymer, which has been used in this study, differed from the aluminum wheel. Khalil et al. [118] studied the effect of different blade heights of rotors on the performance of a micro-scale axial turbine. Different parts of this system, with respect to the exploded assembly drawing, are shown in Figure 4a. In this study, different parts of this system such as reducer, stator, disc, rotor, rotor case, and closing were fabricated by a Polyjet printing technique and using a resin (RGD525) material. The three different blade height sizes of the rotors were 4, 6, and 8 mm (Figure 4b). The experiment was performed in environment temperature and at pressure ratios in the range of 1.2 to 1.75, and rotational speeds of 4000 to 16,000 rpm. According to their results, fabricated rotors with blade heights of 4, 6, and 8 mm were able to produce power up to 630.75, 694.1, and 796.89 W, respectively, at an expansion ratio around 1.75 and through the rotational speed of around 16,000 rpm.

## 4. Structural Stress Analysis

Numerical analyses for parts designed with consideration of working conditions have been one of the most critical steps in the investigation of materials [119]. Regarding impellers there are no exceptions to this, and numerical evaluations have always been one of the vital steps in the investigation of materials for this component. In other words, numerical analyses can be considered as a tool to balance performance and reliability during the development and design of the products [120]. 

In this section, numerically analyzing the centrifugal impellers and rotors of compressors will be discussed. As mentioned, properties such as modulus of elasticity, thermal expansion, fracture toughness, fatigue strength, thermal conductivity, specific heat capacity, corrosion resistance, and thermal stability have been among the parameters considered in the selection of materials for the fabrication of impellers and rotors of compressors [121]. We should mention that centrifugal stress due to rotational forces, bending stress due to fluid pressure and change of momentum, and thermal stresses due to thermomechanical load are the properties that were considered as effective factors in the simulations. So, by examining the materials under the mentioned forces, the authority of the material will be assayed in simulation [122].

Structural analysis is the most common application of finite element analysis which allows for the investigation of different types of loads, including stress, strain, deformation, and so on. The linear structural static equations are as follow [123]:

(1) $$\frac{\partial \sigma_{x}}{\partial x}+\frac{\partial \tau_{xy}}{\partial y}+\frac{\partial \tau_{xz}}{\partial z}+F_{bx}=0$$

(2) $$\frac{\partial \tau_{yx}}{\partial x}+\frac{\partial \sigma_{y}}{\partial y}+\frac{\partial \tau_{yz}}{\partial z}+F_{by}=0$$

(3) $$\frac{\partial \tau_{zx}}{\partial x}+\frac{\partial \tau_{zy}}{\partial y}+\frac{\partial \sigma_{z}}{\partial z}+F_{bz}=0$$

where *σ* represents the normal stress, *τ* shows the shear stress, *Fbx*, *Fby* and *Fbz* are the body forces per unit volume acting along the directions *x*, *y*, and *z*, respectively. 

Centrifugal force per area of the blade appears as centrifugal stresses, which can be written generally as follows:

F_C_ = mrω^2^ (4)

where F_C_ represents the centrifugal force, ω is a rotational speed, r and m show the radius and mass of the considered section, respectively.

In the thermal condition, control equations for linear elastic and isotropic three-dimensional solid materials to considering the thermal load are as follows [124]:

(5) $$\varepsilon =D^{-1}\cdot \sigma +\alpha \cdot \Delta T$$

(6) $$\Delta T=T-T_{\mathrm{Ref}}$$

where *ε*, *σ*, T_Ref_, D and *α* are total strain vector, stress vector, referenced temperature, material elastic stiffness matrix and matrix of thermal expansion coefficient, respectively.

Fluid-structure interaction (FSI) is the approach that can be used for structural examination of the impeller. In this method, the effect of the fluid dynamics on the structural mechanics of the impeller based on computational fluid dynamics (CFD) and structural finite element analysis (FEA) can be analyzed [125]. Solving the Reynolds-averaged Navier–Stokes (RANS) equations has been one of the most used methods in CFD [126]. The equations are as follows [124]:

(7) ∇(ρV)=0

(8) ∇(ρVV)=ρf−∇p+∇T

(9) ∇(ρhV)=ρf·V+∇T·V−∇q

where V, *f*, p, T, h and q are velocity vector of the fluid, body force vector per unit mass, pressure, viscous stress tensor, volumetric enthalpy and heat flux vector, respectively.

**Figure 5:**
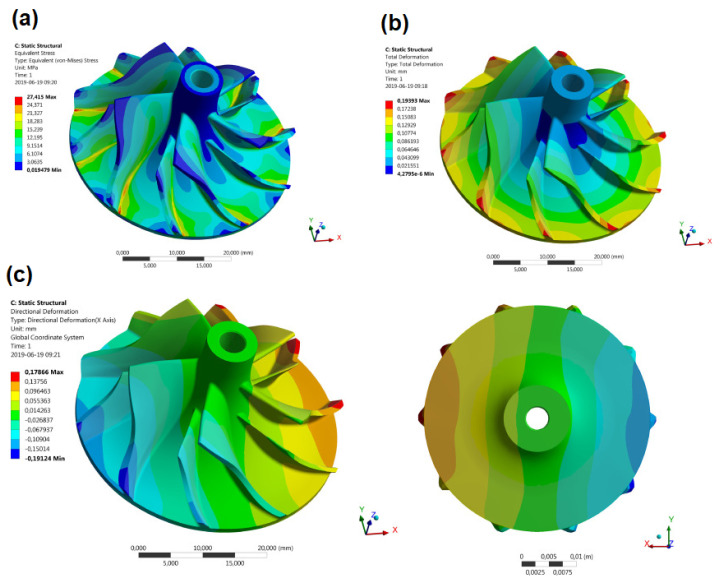
(**a**) stress distribution, (**b**) deformation distribution (**c**) deformation in the X as isometric bottom view on the rotor (reprinted with permission from [9]).

Andrearczyk et al. [9] investigated the plastic wheel in turbocharger application. In this experiment, the rotor has been fabricated by the MultiJet 3D printing method with a diameter of 42.5 mm. For calculation strength analyses, rotational speed and temperature were sets of 100,000 rpm and 55 °C, respectively. According to their results, the maximum deformation and the maximum stress on the impeller geometry were 192 μm and 27 MPa, respectively. Figure 5 shows the stress and deformation results. Also, the performance of the compressor was simulated based on solving RANS by ANSYS to achieve the compressor performance map. Their results showed the streamlines in a relative reference frame, in which the rotational speed and mass flow rate were 200,000 rpm and 0.09 kg/s, respectively.

Kar et al. [18] studied a polymeric impeller in centrifugal pump applications. The impeller had a diameter of around 5.08 cm and has been fabricated by polyetherimide. Structural stress analysis was performed by finite element analysis (FEA) under maximum centrifugal conditions with respect to the rotational speed of 72,000 rpm and gravitational load. Their results showed that the maximum stress on the impeller was 5.45 MPa.

Isaias et al. [11] investigated a different polymer, composite and metal impeller together with the aim of evaluating the feasibility of developing a simplified turboexpander. Calculations under different loadings were examined in this study. Full load conditions, a rotor blocked, which is full flow and being supplied with the rotor stopped, and 27% over-speed due to the consideration of international standards were the conditions which were considered. The rotational speed was 36,000 rpm and the diameter of impeller was 49 mm. Their results showed that the obtained stress showed high sensitivity to rotational force and pressure loading. The equivalent stress on the PEEK impeller in different conditions of loading has been performed and according to their results the maximum stress on the blade was 10 MPa. Also, the value of the factor of safety for aluminum, PEEK reinforced with 30% glass fiber and ABS were 19.92, 22.25 and 13.32, respectively, with an analysis of 27% over-speed. These values were greater than the minimum requirement. Considering their simulation, additional stress caused by temperature was analyzed. The simulated efficiency was 86%, 0%, and 84% for the full load, rotor blocked and 27% over-speed situations, respectively.

## 5. Performance Evaluation

An accurate performance evaluation of the impeller in the pump, compressor or turbine is essential due to it confirming the ability of the machine to respond to working conditions, as well as the correct energy consumption. In this regard, performance investigation is possible through the analysis of work done on working fluid. In general, inlet temperature, inlet pressure, discharge temperature, discharge pressure, rotational speed, differential pressure across flow meter (or pitot traverse), temperature and pressure at the flow meter are among the measurements to determine the machine performance [127,128]. In addition, investigation of vibration has been considered to confirm the dynamic performance. Furthermore, the tribological behavior of the impeller under working conditions is another parameter that should be considered. Depending on the application, such as the pump or compressor, the environment of the test bench would be different [129]. We should mention that performance tests will be performed to achieve the compressor map, which represents the corrected flow versus the pressure rise at various aerodynamic speeds [130].

### 5.1. Pressure Measurements

Measuring the pressure is essential to investigating the performance of the system. So, during the experiment input and output pressure are measured by a pressure gauge. Depending on the kind of pressure being measured such as static, or dynamic pressure, the installation place of the gauge can be different. For example, output pressure can be measured by a pressure gauge installed along the discharge path. 

Mojaddam and Torshizi [131] studied the impeller hub and shroud of a radial flow compressor by implementing different meridional contours on the same impeller characteristics. Evaluation of performance was performed by considering the pressure ration from a compressor inlet to a diffuser outlet. According to the comparison of compressor performances for both cases, in circular and elliptical hub and shroud curves, pressure ratio and the isentropic efficiency in different mass flow rates at a fixed rotational speed have been shown. The difference in pressure ratio was minimal at low rotational speed, so that the maximum difference was 1.4% at the highest mass flow rate. For their design rotational speed differences were considerable at 3% and at the maximum mass flow rate at approximately 10%. Also at high rotational speed, the pressure ratio was the same. In conclusion, pressure ratio and total-to-total isentropic efficiency for both impellers along with inlet section and vane-less diffuser were selected to evaluate the newly suggested curves. Their results showed that the elliptical curves have acceptable performance in comparison with circular curves.

Li et al. [132] investigated the impact of the blade angle of the plastic impeller on the performance of the centrifugal pump. They analyzed the pressure fluctuation at the outlet of the impeller. According to their results increasing the outlet angle and inlet angle played a key role in machining the optimal performance. The best results have been achieved for an outlet angle of 35° plastic impeller, in which efficiency and head were 81.02% and 35.80 m, respectively. In general, the optimal performance of the printed impeller has been shown according to the simulation and experimental results.

### 5.2. Mass Flow Measurements

To analyze the system performance and achieve the compressor and pump maps, measurement of the mass flow is essential. This parameter can be measured by a flow meter. Mass flow rate refers to the product of the working fluid density, the cross-sectional area and the flow velocity [133]:

(10) $$\dot{m}=\rho \;A\;V$$

In the case of compressors, the corrected mass flow rate can be calculated by the following equation [134]:

(11) $${\dot{m}}^{*}=\dot{m}\;\left[\frac{P_{ref}}{P_{in,\;0}}\right]\sqrt{\frac{T_{in,\;0}}{T_{ref}}}$$

where *T_in_*, 0 and *P_in_*, 0 are the total temperature and total pressure at the compressor inlet, respectively, and *T_ref_* and *P_ref_* are 288.15 K and 1 atm, respectively.

**Figure 6:**
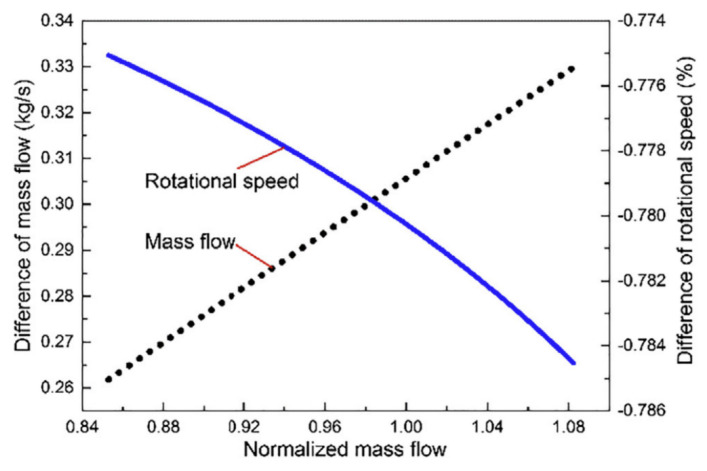
Differences between saturated humid and corresponding dry air (reprinted with permission from [135]).

Sun et al. [135] evaluated the influence of humidity on the performance of a centrifugal compressor. Their results showed that pressure ratio and peak isentropic efficiency have been decreased by increasing the humidity. Also, the variation in performance was analyzed by measuring the mass flow in different rotational speeds in humid and dry air conditions. The mechanism of influence on performance was analyzed by measuring the mass flow in dry and humid conditions. According to their results, at the same rotational speed, the mass flow of humid air was smaller than that of dry air. Figure 6 shows the differences between saturated humid air at 100% design rotational speed and its corresponding dry air.

### 5.3. Vibration Analysis of Impeller

The importance of analyzing the vibration of impellers has been mentioned in a lot of studies [136]. Increasing cyclic stress and fatigue failure, collision of the rotor with stationary parts, seized bearings, vibrating force transmission to stationary parts, and induced vibration of peripheral units have been among the problems due to rotors vibration. The natural frequency from the impeller or rotor vibration by the 3-D finite element method has been analyzed by considering the difference between the inertial coordinate system fixed to the stationary side and the rotational coordinate system fixed with the rotor. Due to the symmetry of the impellers with respect to the center of rotation, the analysis of rotating structures by 3-D finite elements is considered cyclic symmetry [137]. 

**Figure 7:**
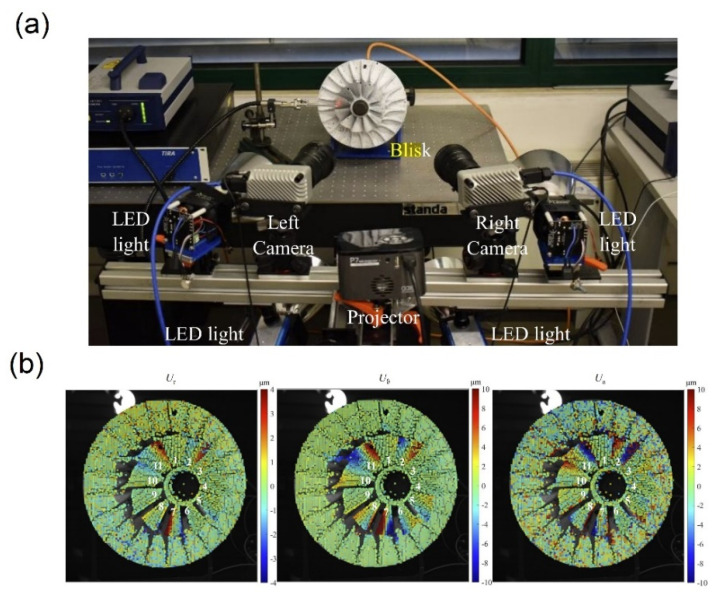
(**a**) Optical setup of the stereo-digital image correlation measurement and (**b**) Displacement maps for the 6239 Hz excitation frequency (reprinted with permission from [140]).

Vibration measurements can be performed by a vibration analyzer [138] or digital image correlation (DIC) by cameras [139]. In this way, amplitude in the different range of frequency or displacement magnitude against the time in a certain frequency is achievable. Neri et al. [140] described a measurement system to investigate the impeller damping (Figure 7a) by measuring the excitation force during the test with respect to the response and the load amplitude. The vibrational measurements in the high-frequency range of 2568 Hz, 6239 Hz and 6357 Hz showed a harmonic response and frequency values in combination with the low response amplitudes, in the range of 10 µm. Their results in the high frequency of 6239 Hz in the cylindrical reference frame coordinates showed a smooth map for all three directions (Figure 7b).

Mousmoulis et al. [141] studied the vibration of pump impeller considering the important effect of the cavitation in the steady and dynamic operation of a pump. In this regard, vibration measurements for the inception of cavitation have been performed in the frequency range of 5–10 kHz. Given that the impellers with different geometries have been considered, the lower incidence angle and the use of splitter blades showed the milder noise and vibration characteristics through the entire Thoma number range tested. Also, they mentioned that increases vibration band power at part load conditions can be due to the increasing turbulence intensity and the backflow cavitation mechanism.

### 5.4. Tribology Behavior Analysis

Tribology can be defined as a science of surfaces in contact with each other and consists of friction, lubricant and wear [142]. Considering the interaction between working fluid and impeller, investigation of wear resistance and friction can play an important role in the efficiency of the machine. So, analyzing the tribology behavior, which is the science of interacting surfaces in relative motion, has a high position [143]. For example, different friction on the impeller caused by various viscosity of fluids impacts the performance of the system. On the other hand, the viscosity of fluid has an effect on the friction losses and can change the performance, such that increasing the fluid viscosity causes a reduction in performance [144,145]. So, the viscosity of the fluid can change the characteristic curve of the systems. Considering the characteristic curves, viscosity correction factors can be obtained and viscosity correction factor can be defined through the η/ηwater, where η and ηwater are efficiency of viscous fluid and water, respectively [145]. 

The wear of the impeller during working is another important parameter that should be considered in analyzing the performance of systems. Wear is defined as the removal of material from a solid surface caused by friction or impact. Jiang [19] studied the wearing properties of the fabricated impellers of PLA, ABS and VeroGray by a 3D printing method. The wear test was performed in different concentrations of erodent material for 110 h. During the experiment after every 5 h, the weight loss of the impellers to calculate wear rate was measured. According to their primary results, the VeroGray impeller fabricated by Polyjet 3D printer highly reduced the experimental time and cost and was chosen as the impeller for analysis of the wear test. VeroGray impeller at a rotational speed of 1200 rpm and in presence of 5% concentrations of erodent during the 110 h showed a mass loss percentage around 8.08%.

**Figure 8:**
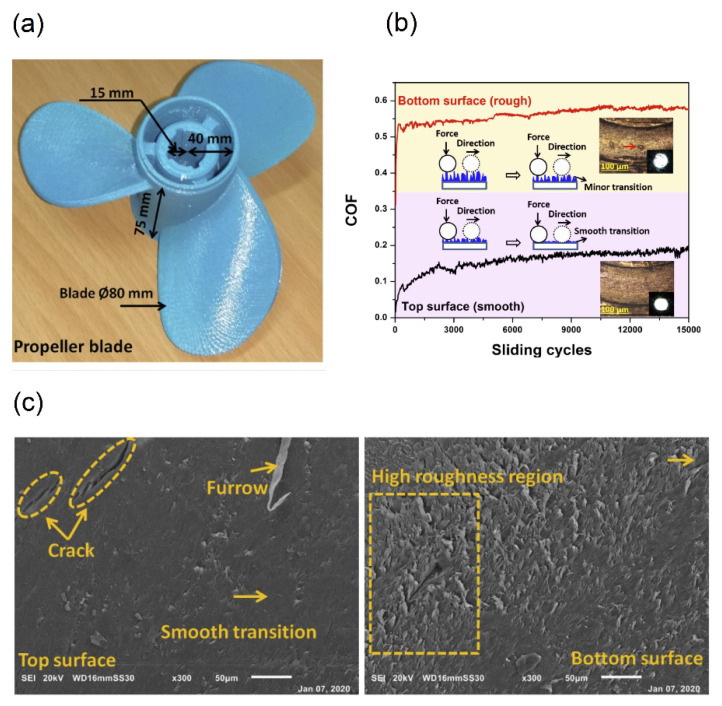
(**a**) 3D printed propeller blade, (**b**) Friction performance of PLA sample at the top and the bottom surface and (**c**) wear microstructure of PLA samples (reprinted with permission from [14]).

Upadhyay et al. [14] investigated the tribotechnological and mechanical properties of PLA propeller blades in a marine application. They studied the friction and wear behavior with its sliding wear mechanism, due to the importance of tribotechnological, and studied soft and hard interfaces with degradation properties. Figure 8a shows the PLA-propeller blades and (b) represents the result of coefficient of friction (COF) of PLA sample for smooth and rough surfaces at a relative humidity of 40%. The average COF of PLA sample at the top and the bottom surface was 0.158 and 0.56, respectively, with respect to the immersion time, which was 30 days in seawater. The wear microstructure at the top and bottom surface of the PLA sample was shown in Figure 8c. Sliding at the polymer sample’s top surface provides a smooth transition to the ball material due to low surface roughness. According to their results, they justified the immediate suitability of 3D printed PLA parts for practical marine application by designated tests of sliding and degradation.

## 6. Conclusions

Studies have shown an increase in efficiency and effectiveness through the use of polymers. Among thermoplastic polymers and composites, PEEK and PPS with and without reinforcements were introduced as suitable options for the fabrication of impellers. On the other hand, in the case of turbochargers, the use of resins to make the wheels was recognized as a suitable option. Conventional impeller manufacturing, injection molding and additive manufacturing were the common methods in producing the impellers. In general, the softness of the surface obtained by various methods had an acceptable level for use in the field of compressors and pumps. However, the time-consuming nature of the 3D-printing method, for example, compared to the injection method will require further improvements. However, among the 3D printing methods, the SLA and MultiJet printing methods can provide a more appropriate surface than other methods due to the production process. High anisotropy in FFF samples is also a challenge in using this method in the construction of impellers, which requires more study. In injection molding and conventional machining methods, they can be examined in order to fabricate the geometries with less complexity compared to additive manufacturing. Fluid-structure interaction, as well as performance evaluations to analyze the impeller, are key steps in the final evaluation of impellers. In conclusion, the use of polymers and polymer composites were promising options as alternatives to metals in some applications. Limitations of plastic impellers have also been identified, including the potentially shorter lifespan of blades (compared with its metal counterpart) when moisture or impurities are present in the working fluid and need more research.

## Figures and Tables

**Table 1:** Physical and mechanical properties of polymers used in the fabrication of impellers.

	PLA	ABS	PPS	PETG	PEEK
Glass transition temperature (°C)	56–63	102–115	75–85	49–75	142.85
Melting temperature (°C)	125–178	-	285	-	342.85
Modulus (GPa)	1.03–4.0	1.8–2.39	3.9–4.1	0.9–1.6	3.6
Tensile strength (MPa)	51.7–80.9	42.5–44.8	79	44.12–57	107
Ref.	[30]	[31]	[32]	[33,34]	[35]

## Data Availability

Not applicable.
